# RAPPID-M: A Mix-and-Measure Bioluminescent Sandwich Immunoassay Based on Generic Antibody-Binding Protein M

**DOI:** 10.1021/acssensors.5c02450

**Published:** 2025-10-10

**Authors:** Anna Swietlikowska, Eva van Aalen, Max Bossink, Laura van Weesep, Maarten Merkx

**Affiliations:** † Laboratory of Chemical Biology, Department of Biomedical Engineering, Eindhoven University of Technology, PO Box 513, Eindhoven 5600 MB, The Netherlands; ‡ Institute for Complex Molecular Systems (ICMS), Eindhoven University of Technology, PO Box 513, Eindhoven 5600 MB, The Netherlands

**Keywords:** immunoassay, bioluminescence, protein engineering, antibody conjugation, biosensor

## Abstract

Homogeneous immunoassays that allow direct in-sample detection of biomarkers provide an attractive alternative to traditional heterogeneous immunoassays that require multiple washing and incubation steps. We previously reported the development of RAPPID, a bioluminescent sensor platform that uses split luciferase complementation to enable sandwich immunoassays directly in solution. Although RAPPID provides many benefits over traditional heterogeneous immunoassays, it requires protein G-mediated photoconjugation of split luciferase fragments to the Fc part of IgG monoclonal antibodies. Here, we report a new generation of RAPPID sensors (RAPPID-M) that do not rely on photoconjugation of protein G, but instead use a generic antibody-binding domain derived from protein M. The use of protein M substantially expands the application of RAPPID to include protein G-incompatible but analytically important mouse IgG1 antibodies, as well as scFv and Fab antibody fragments. Moreover, binding of protein M is found to be essentially irreversible, abolishing the need for photo-cross-linking. RAPPID-M thus improves upon the original RAPPID platform by the mix-and-measure-type assembly of antibody-split-luciferase conjugates and its compatibility with a broader scope of antibody types without compromising and even improving the analytical performance.

## Graphical abstract

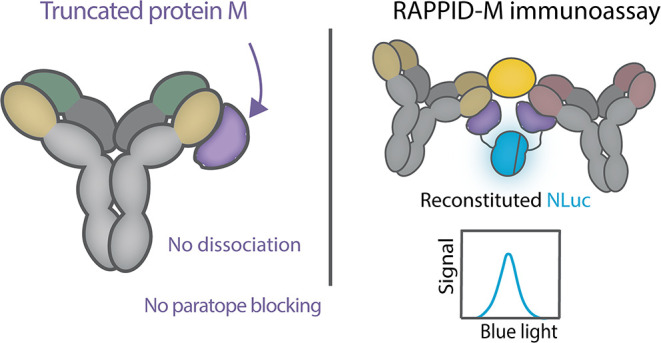

Enzyme-linked immunosorbent assays (ELISAs) and other immunoassays are widely applied in both research and clinical laboratories.^1^ However, classical heterogeneous immunoassays involve a complex workflow of antibody immobilization, time-consuming incubations, and multiple washing steps. In addition, each new immunoassay requires careful optimization of immobilization and assay conditions to minimize background binding. An attractive alternative is provided by biosensor proteins that allow single-step immunoassays directly in solution. The short sample-to-answer time and limited sample handling allow these homogeneous immunoassays to be performed directly at the point of need. Homogeneous immunoassays based on bioluminescence are attractive in this respect because bioluminescence does not require external illumination, enabling sensitive detection of biomarkers in complex matrices using a standard smartphone or digital camera as the reader device.^2–4^

In recent years, we and others have developed several homogeneous bioluminescent immunoassay platforms that either rely on proximity-driven complementation of a split luciferase or modulation of intramolecular split luciferase complementation.^5–14^ A particularly versatile format is RAPPID (Ratiometric Plug-and-Play ImmunoDiagnostics),^5,6^ a homogeneous sandwich immunoassay in which the split NanoLuc fragments,^15^ LargeBit and SmallBit, are conjugated to two detection antibodies. Binding the two antibody conjugates to the analyte brings them into close proximity, which enables complementation of the split luciferase fragments and the generation of blue light. RAPPID sensors typically display a high dynamic range, showing an increase in luminescence of 100-fold or more and a low, pM limit of detection (LOD). Moreover, RAPPID does not require complicated protein engineering, as it uses protein G-mediated photoconjugation to generate well-defined antibody conjugates directly from commercially available antibodies.^16^ The protein G domain contains a non-natural, photo-cross-linkable amino acid (*p-*benzoylphenylalanine, pBPA), which forms a covalent bond with a methionine residue present in the Fc part of many mammalian IgG antibodies upon UV exposure.^17^ Photo-cross-linking typically results in a mixture of non-, one-, and two-conjugated antibodies, which can be improved by using a tandem of two protein G domains fused via a semiflexible linker.^18^ While efficient protein G photo-cross-linking has been reported for human IgGs and several types of mammalian IgGs (rat, mice, goat), protein G photo-cross-linking is incompatible with mouse IgG1, an important subclass of diagnostic antibodies, most sheep antibodies, and avian IgY. Furthermore, since protein G binds between the CH2 and CH3 domains of the Fc part,^16,19^ RAPPID is incompatible with antibody fragments and other antibody formats lacking the Fc domain.

In this work, we report the development of a new generation of RAPPID sensors that do not require photo-cross-linking to the Fc part but instead use a generic antibody-binding domain derived from protein M (RAPPID-M). Protein M is a protein displayed on the surface of human mycoplasma (*Mycoplasma genitalium*) that binds with low nM affinity to the κ and λ light chains of human IgG, IgA, and IgM as well as murine, rabbit, goat, bovine IgG, and chicken IgY.^20–24^ Binding of wild-type protein M has been shown to sterically block the antigen-binding site via its α-helical C-terminal domain. Here, we show that a truncated version of protein M (pM440) lacking the antigen-blocking domain (residues 441–468) retains the high binding affinity and low dissociation rate of the parent protein M, without interfering with antigen binding. Replacing the photo-cross-linkable protein G domains with pM440 in RAPPID enables the formation of noncovalent, yet very stable antibody-split luciferase complexes by simply mixing the components (Figure 1). When using the same analyte and detection antibodies, RAPPID-M improves upon the already high dynamic range of the original protein G-based RAPPID (RAPPID-G). Despite the lack of covalent cross-linking, the RAPPID-M system showed no appreciable dissociation and was stable over several days. Furthermore, we demonstrate the successful application of RAPPID-M using antibodies incompatible with protein G photo-cross-linking, such as mouse IgG1, scFv, and Fab antibody fragments.

**Figure 1:**
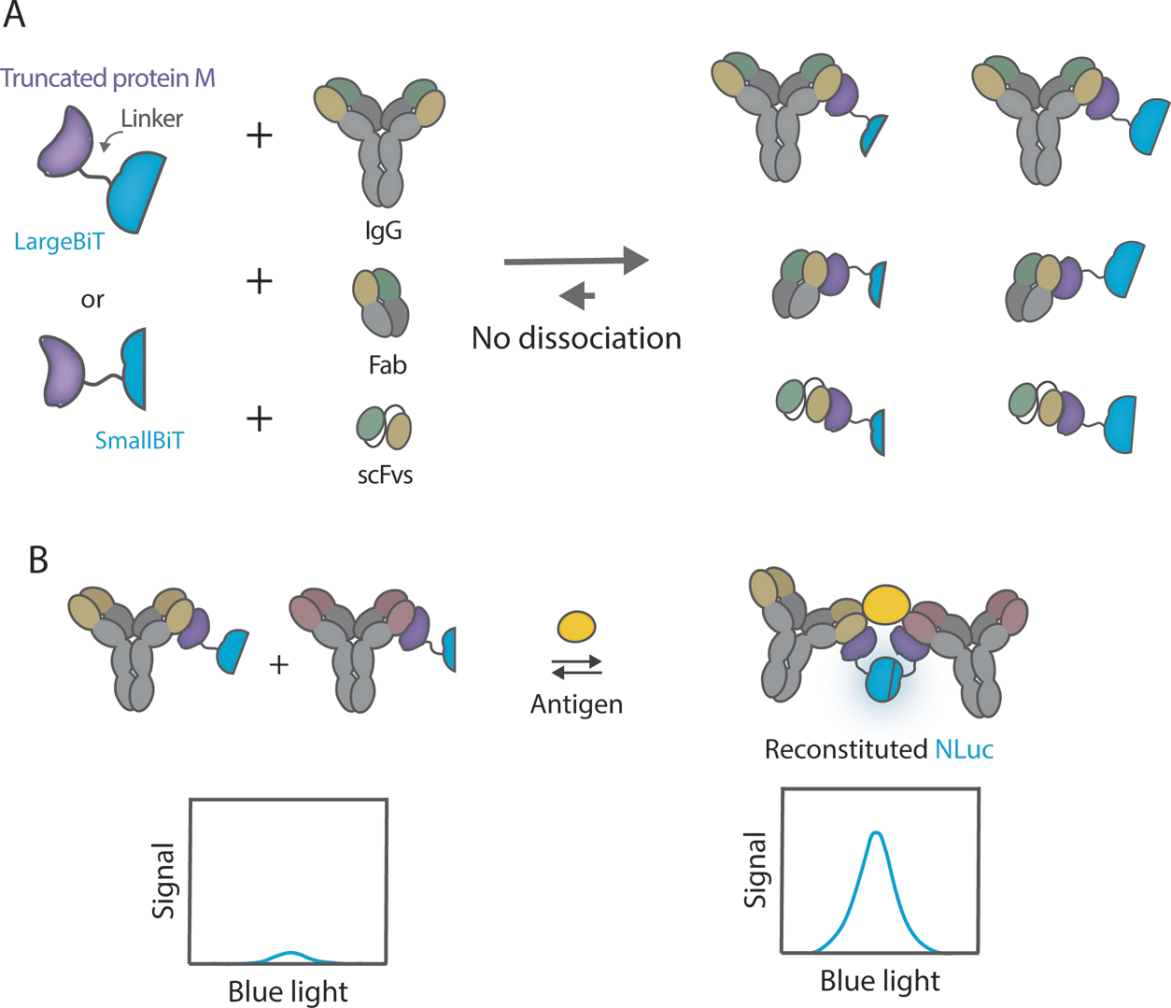
Overview of the RAPPID-M sensor platform. (A) Overview of the RAPPID sensor constructed with protein M440 (pM440). The ability of protein M to bind to a light-chain variable domain allows the labeling of IgGs, fragment antigen-binding regions (Fabs), and single-chain variable fragments (scFvs). (B) Homogeneous RAPPID immunoassay applying pM440. Binding of antigen-specific antibodies, complexed with split luciferase fragments via pM440, to the distinct epitopes of the antigen results in a high local concentration of the split luciferase domains, the reconstitution of the active luciferase, and subsequently the emission of a bioluminescence signal.

## Results and Discussion

### Characterization of Truncated Protein M and Its Interaction with IgGs

The binding of wild-type protein M to antibodies has been reported to sterically block the binding of protein antigens while still allowing the binding of sterically less demanding small molecules.^21^ Previous work showed that the removal of the C-terminal residues 441–468 restored accessibility to the antigen-binding site to protein antigens, but the effect of binding of the truncated protein M variants, protein M440 (pM440) and protein M420 (pM420), on the affinity and kinetics of antigen binding was not extensively characterized.^20,21^ In our hands, pM420 was found to be prone to aggregation (results not shown), but pM440 could be successfully expressed in *Escherichia coli* and purified in high yield (38 mg/L; Figure S2a). The removal of the C-terminal antigen-blocking domain did not affect the thermodynamic stability of protein M, as the melting temperature of pM440 obtained by using differential scanning fluorimetry (DSF) was found to be similar to that of full-length protein M (Figure S3). Analytical size-exclusion chromatography (SEC) revealed that pM440 forms a monomer and confirmed its ability to form a stable complex upon the addition of an antibody (Figure S4).

An interesting property of full-length protein M is that the dissociation of the protein M–antibody complex has been reported to be extremely slow, which may alleviate the need for covalent cross-linking.^22,24^ To establish whether this feature is retained in truncated protein M, we used surface plasmon resonance (SPR) to study the association and dissociation kinetics for the interaction between pM440 and Adalimumab, a TNFα–binding therapeutic antibody (Figure 2a). Minimal dissociation was observed over a period of 1 h, which is consistent with a *k*_diss_ of ≤ 6.4 × 10^–6^ s^–1^ (Figure S5) and similar to that previously reported for full-length protein M.^22^ Please note that *k*_diss_ represents an upper limit, which, when combined with the *k*_ass_ of 4.3 × 10^4^ M^–1^s^–1^, corresponds to a *K*_D_ ≤ 0.15 nM. These SPR measurements show that the remarkable stability of the protein M–antibody interaction is retained in protein M440.

**Figure 2:**
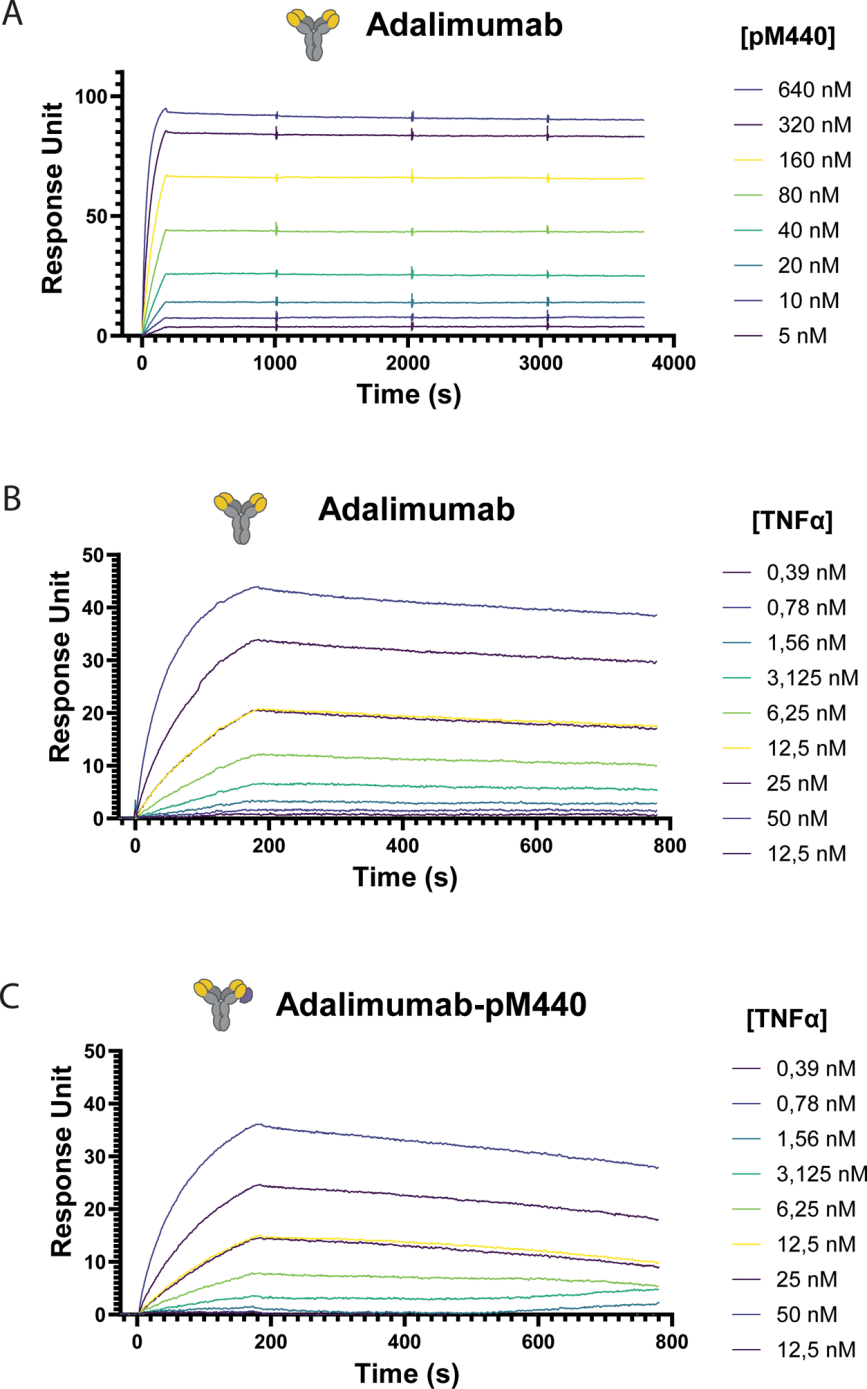
SPR analysis of pM440–antibody-binding kinetics and the influence of pM440 binding on the antigen–antibody interaction. (A) SPR analysis of pM440 binding to Adalimumab. The blank-subtracted sensorgram presents the Adalimumab antibody immobilized on the protein G-functionalized chip and the binding to various concentrations of protein M440. Data fits are shown in Figure S5B,C. SPR analysis of the interaction between Adalimumab and TNFα in the absence (B) and presence (C) of protein M440. To immobilize the antibody, Adalimumab was either directly applied to the SPR chip or precomplexed with a 4 molar excess of pM440. The data fits are shown in Figure S6.

Next, we performed SPR measurements to study whether binding of pM440 affects the antibody–antigen interaction, as this could attenuate the sensitivity of immunoassays. Adalimumab (1 μM) was precomplexed with a 4-fold molar excess of pM440 to ensure the saturation of both antigen-binding domains and the formation of a 2:1 pM440–antibody complex. Following overnight incubation, the Adalimumab–pM440 complex was immobilized on a protein G-functionalized chip and compared to Adalimumab in the absence of pM440. Various concentrations of TNFα, ranging from 0.39 nM to 50 nM, were flown over the sensor surface, each followed by a 9 min dissociation step (Figure 2B,C). Fitting of the sensorgrams revealed a small decrease in *k*_ass_ from 2.6 × 10^5^ to 1.6 × 10^5^ M^–1^s^–1^ and 2-fold increase in *k*_diss_ from 2.2 × 10^–4^ to 4.6 × 10^–4^ s^–1^ in the presence of pM440. This results in a 3-fold decrease in TNFα affinity, showing that the binding of pM440 has only a minor effect on antigen binding. Similar results were obtained using SPR single-cycle kinetic titration experiments (Figures S7 and S8), yielding a dissociation constant of 0.65 nM and 2.94 nM for the binding of TNFα to Adalimumab and the Adalimumab–pM440 complex, respectively. SPR experiments using a second antibody, the interleukin-6 targeting antibody R508, showed similar results and confirmed that binding of protein M440 did not interfere with binding of the IL-6 antigen (Figure S9).

### Development of RAPPID-M Sensors and Comparison to RAPPID-G

Having established that pM440 does not significantly interfere with analyte binding and retains very slow dissociation kinetics, fusion proteins were constructed between pM440 and either SmallBit or LargeBit. A semiflexible linker was introduced between pM440 and the NanoBit components that was long enough to cover the distance between the two antibodies in a sandwich complex.^5^ Similar to the original RAPPID sensor, an affinity of 2.5 μM was chosen for the interaction between SmallBit and LargeBit.^15^ This affinity prevents complex formation in the absence of antigen, but is high enough for luciferase complementation upon the formation of a sandwich complex.^1^ The pM440-LargeBit (pM440-LB) and pM440-SmallBiT (pM440-SB) proteins were recombinantly expressed in *E. coli* and purified by Ni^2+^ affinity and Strep-Tactin affinity chromatography. Final yields were 12 mg/L for pM-SB and 13 mg/L for pM-LB with >95% purity (Figure S2B,C).

To provide the proof of principle, we first developed a RAPPID-M sensor for IL-6 and compared its performance to that of a RAPPID-G assay using the same commercially available anti-IL-6 antibodies R508 and mhK23. R508 and mhK23 are rabbit anti-IL-6 and mouse chimeric monoclonal antibodies, respectively, that are compatible with protein G photo-cross-linking, enabling direct comparison between RAPPID-G and RAPPID-M. The RAPPID-M sensor components were prepared by simply mixing R508 with pM440-LB and mhK23 with pM440-SB (1:2 ratio, antibody to pG-LB/SB), followed by overnight incubation at 4 °C. For RAPPID-G, R508 and mhK23 were incubated for 45 min with a 2-fold excess of pG-LB and pG-SB, respectively, followed by 10 min photo-cross-linking through irradiation with a 365 nm light. The conjugation mixtures were not further purified and used directly in the RAPPID-G assay. Figure 3A,B shows the IL-6 titration experiments for RAPPID-M and RAPPID-G, respectively. In both cases, bioluminescence was measured after 2 h incubation of 0.1 nM antibody-LB and 0.5 nM antibody-SB with various concentrations of IL-6. The performances of the RAPPID-M and RAPPID-G assays are comparable, both measuring IL-6 at concentrations as low as 1 pM and showing a large increase in bioluminescence up to ∼1 nM of IL-6, with a subsequent decrease at higher IL-6 concentrations due to the Hook effect.^5^ While the maximal signal of RAPPID-G was 50% higher than that for RAPPID-M, the relative signal increase was 3-fold higher for RAPPID-M (413-fold compared to 133-fold), caused by a ∼5 times lower background of RAPPID-M. To investigate the origin of the lower background, we also measured the bioluminescent activity of the RAPPID-M and RAPPID-G sensor components, both separately and as 1:5 mixtures in the absence of antigen (Figure 3C). These experiments show that the higher background bioluminescence observed for RAPPID-G is mainly due to luciferase activity of the LB domain when conjugated to protein G, whereas the fusion of LB to pM440 shows no residual bioluminescence activity. The residual luciferase activity observed for pG-LB may result from transient interactions between LB and protein G. Such a “pseudo-luciferase” activity has recently also been reported for other, nonenzymatic proteins with apparently suitable hydrophobic pockets.^25,26^

**Figure 3:**
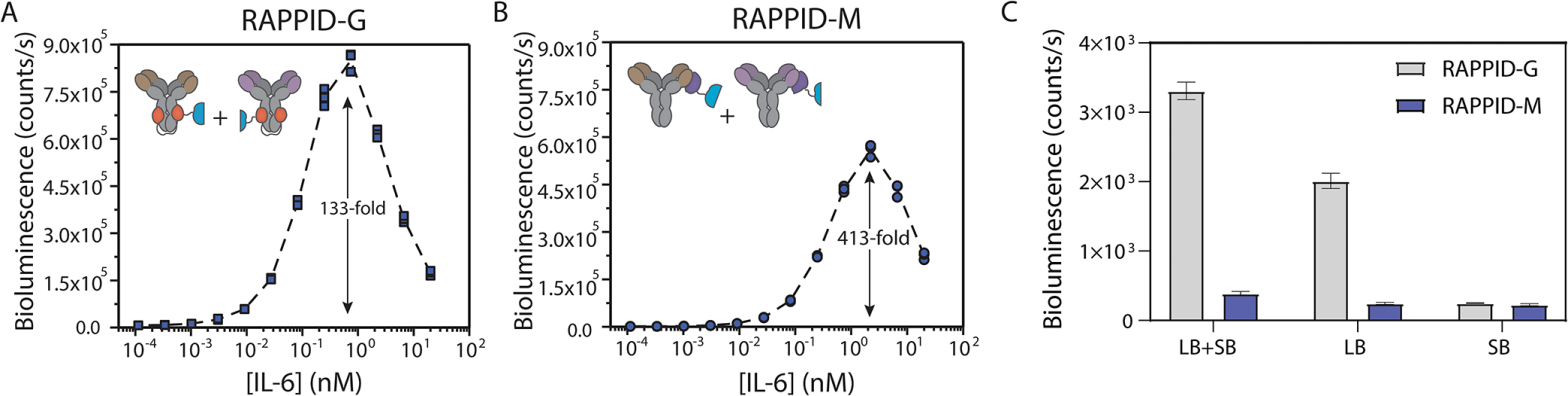
Comparison between RAPPID-M and RAPPID-G. (A, B) Dose–response curves of the RAPPID-G and RAPPID-M assays for detecting IL-6, respectively. (C) Background bioluminescence of (combinations of) various RAPPID-M and RAPPID-G components, 1 nM R508-LB or 0.5 nM mhK23-SB (or combined).

For practical reasons, the antibody–protein M complexes were prepared by overnight incubation at 4 °C, but the SEC experiments show that complex formation should be complete within 15 min at low μM concentrations of antibody and pM440-fusion proteins (Figure S4). Indeed, very similar assay performance was observed when the RAPPID-M assay was performed within 30 min after mixing of the antibodies with pM440-LB and pM440-SB (Figure S10), validating the mix-and-measure concept of the RAPPID-M sensor. To test the performance in a more complex matrix, we compared the performance of RAPPID-M in the absence and presence of 10% human serum (Figure S11), and similar titration curves were obtained with a LOD of 2 pM and a maximum signal at 2 nM of IL-6. A ∼10 times higher background was observed in 10% serum, which attenuated the change in luminescence intensity from 535- to 55-fold. While the latter still represents a very robust increase in signal, the higher background activity could affect the LOD in more complex media such as undiluted plasma. Depending on the origin of the increased background activity, this may require attenuating the interaction between the LargeBiT and SmallBiT domains.

The very low background observed for the RAPPID-M assay in the absence of antigen suggests that the pM440–antibody complexes are stable over the 2 h incubation period, as otherwise exchange would occur between antibodies carrying pM440-LB and pM440-SB fusion proteins. The latter would allow the reconstitution of split luciferase by the binding of pM440-LB and pM440-SB to the Fab domains present in a single IgG antibody, which should result in an increase in background activity. To provide an even more stringent test for the stability of the RAPPID-M system, we also monitored its performance over a period of up to 8 days after mixing the antibody–pM440-LB and antibody–pM440-SB complexes. To do so, assay mixtures were prepared containing 0.2 nM LB–antibody complex and 1.0 nM SB–antibody complexes. Antibody complexes for RAPPID-M and RAPPID-G were prepared as described above, but we also included a variant of the RAPPID-G assay in which the protein G domains were not photo-cross-linked. The latter served as a control to show how domain exchange can influence the assay performance over time. Figure 4B shows the dose–response curves when the assay mixtures were immediately incubated with different IL-6 concentrations for 2 h (day 0). The assay mixture with unconjugated protein G sensors reaches a similar maximal activity, but shows a substantially higher background. As a result, only an 8-fold maximal increase in bioluminescent signal is obtained for unconjugated RAPPID-G, compared to a 109-fold increase for photo-cross-linked RAPPID-G and a 669-fold increase for RAPPID-M. These results confirm the importance of photo-cross-linking for RAPPID-G, as an otherwise substantial exchange of LB-pG and SB-pG results in a high background. To challenge the stability of the complexes, we repeated the IL-6 titration experiments following incubation of the assay mixtures at 4 °C for 4 and 8 days. The dose–response curves obtained after 4 (Figure 4C,F) and 8 days (Figure 4D,G) were remarkably similar to those obtained on day 0. Only a minor decrease in the ratio between maximal signal and background was observed, with RAPPID-M (354-fold) remaining superior to photo-cross-linked RAPPID-G (71-fold) and non-cross-linked RAPPID-G (4-fold). These results show that even over a period of 8 days, the amount of exchange of pM440-fusion proteins between the two detection antibodies is negligible, demonstrating that using truncated protein M instead of protein G abolishes the need for photo-cross-linking.

**Figure 4:**
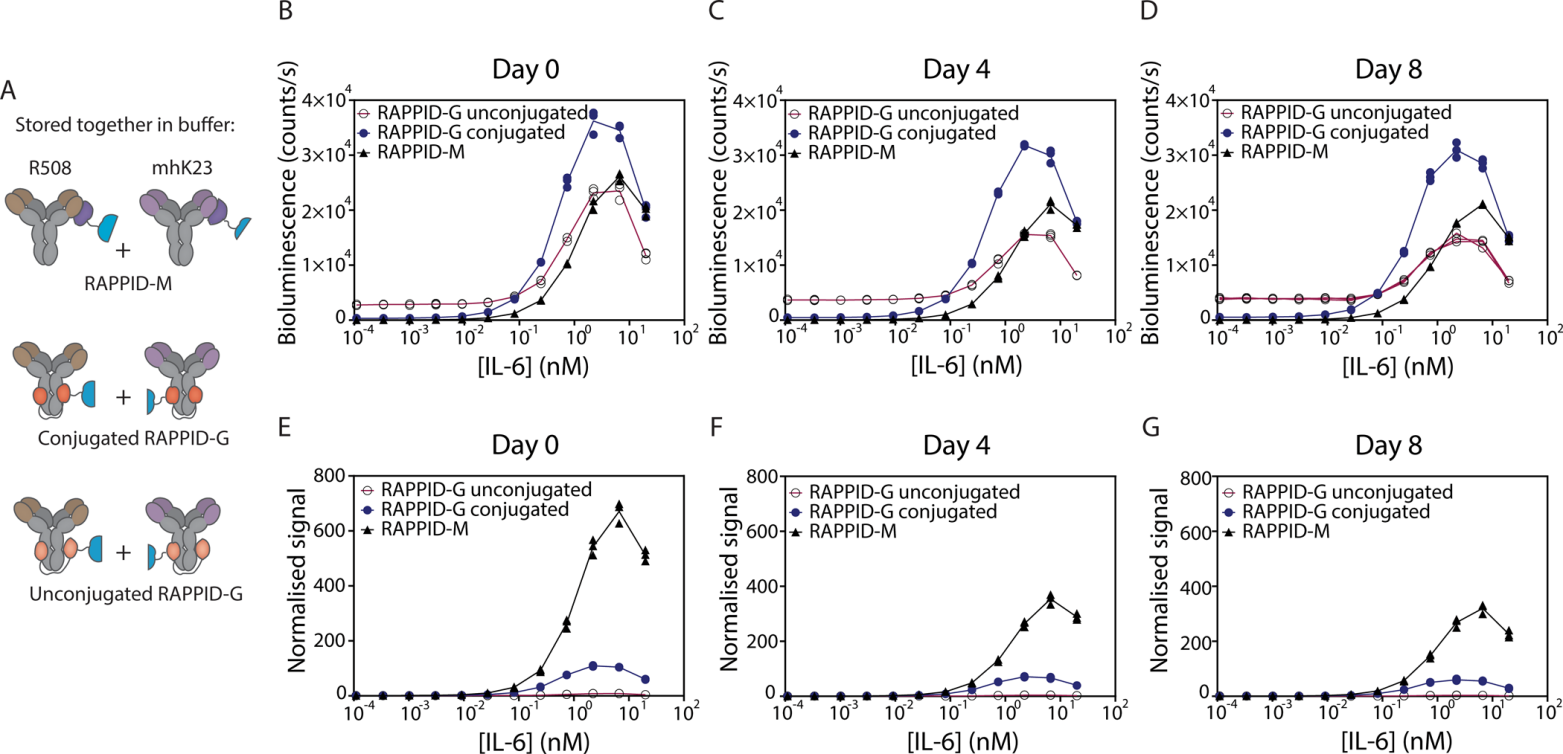
Performance of the protein G and protein M based RAPPID sensors over time. (A) Graphical representation of studied RAPPID sensor mixtures. R508 and mhK23 anti-IL-6 antibodies were complexed with LB and SB via pM440 or via pG (with and without photo-cross-linking). (B–D) Bioluminescence dose response curves of RAPPID sensors over the course of 8 days. (E–G) Normalized data for dose response curves presented in Figure 4B–D, highlighting the higher fold change of RAPPID-M sensor over RAPPID-G.

One of the benefits of RAPPID-M is that it should allow the use of high-affinity mouse IgG1 antibodies, which are commonly used in sandwich immunoassays. We therefore also developed a second RAPPID-M assay for IL-6 using a sandwich pair of mouse IgG1 antibodies, Coat and Detect (a kind gift from Essange). Complexes of pM440-LB and pM440-SB with Detect and Coat antibodies were prepared as described above by overnight incubation at 4 °C of 1 μM antibody with 2 μM pM440-LB or pM440-SB. The assays were performed by incubation of 0.1 nM Detect-LB and 0.5 nM Coat-SB with various concentrations of IL-6 for 2 h. Again, an impressive 371-fold increase in bioluminescent signal was observed upon the addition of IL-6 up to 20 nM (Figure 5A). Importantly, this IL-6 RAPPID-M assay displays a very low LOD, allowing the detection of 29 fM of IL-6 (Figure 5B), a clinically relevant sensitivity that rivals that of the most sensitive heterogeneous immunoassays.^27^

**Figure 5:**
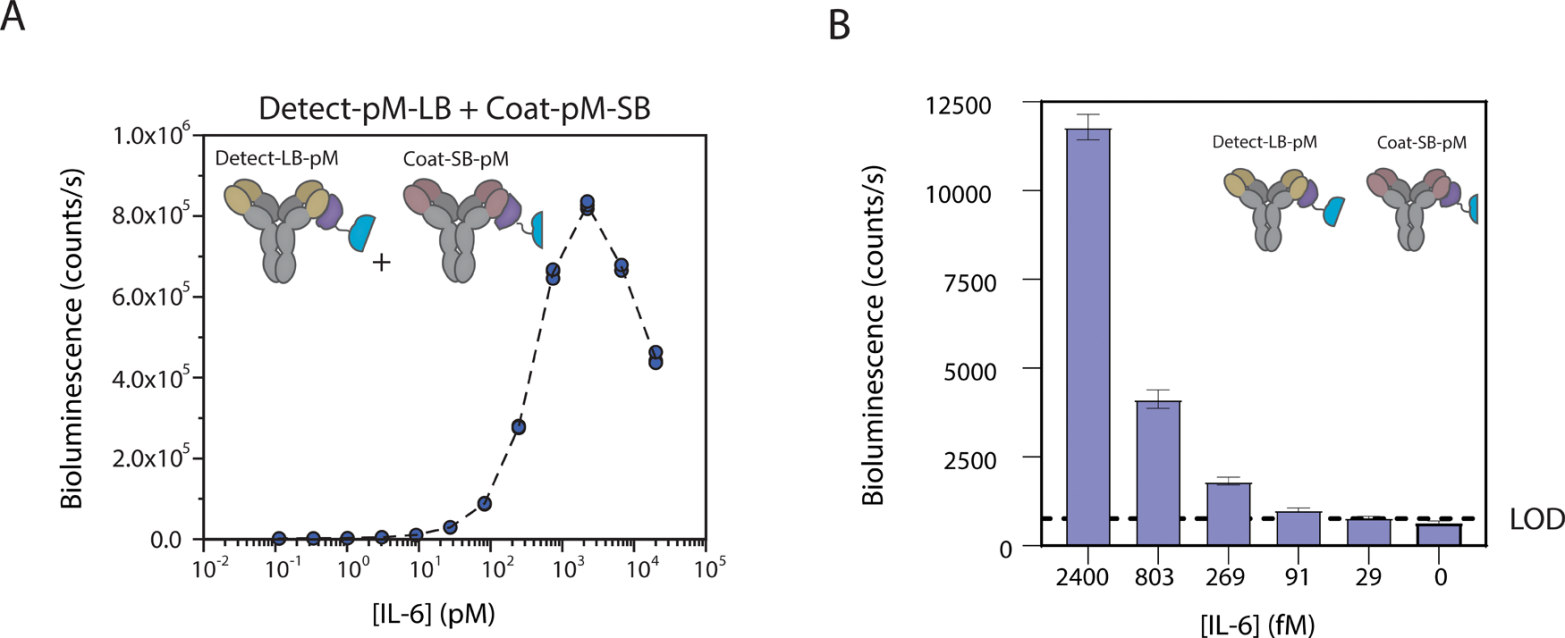
Extension of RAPPID technology to mouse IgG1 antibodies. (A) Dose–response curve of RAPPID-M using the mouse IgG1 antibodies Coat and Detect. (B) Investigation of the limit of detection (LOD) of the IL-6 assay.

### RAPPID-M with Various Antibody Formats

Since protein M binds to the variable domains of antibodies, RAPPID-M should also be compatible with antibody formats lacking the Fc part, such as Fab and scFv fragments. Unlike full-sized monoclonal antibodies that require expression in mammalian cells, Fab and scFv fragments can be produced using relatively inexpensive *E. coli* or yeast expression systems. In addition, Fab and scFv are monovalent binders, which in some cases allow for more homogeneous binding interactions. To demonstrate the compatibility of RAPPID-M with antibody fragments, we developed RAPPID-M assays for anti-Cetuximab and TNFα using Fab- and scFv-binding domains, respectively. Fab fragments of Cetuximab were produced by the proteolytic cleavage of Cetuximab and subsequent purification using a Pierce Micro Fab Preparation kit (Thermo Scientific). The obtained Fab fragments were mixed with pM-LB or pM-SB in a 1:2 ratio and incubated overnight at 4 °C. Using 1 nM of Fab-pM-LB and 1 nM of Fab-pM-SB, a 7-fold increase in bioluminescence activity was obtained upon the addition of increasing concentrations of anti-Cetuximab antibody, with the lowest measured target concentration of 62.5 pM (Figure 6A). Although less impressive in terms of dynamic range than the RAPPID-M IL-6 assays, these results show that the use of protein M does allow extension of the RAPPID assay to Fab fragments. scFv fragments incorporating variable domains of Adalimumab were produced using recombinant expression in *E. coli* (Figure S9). Since TNFα is a homotrimer, the same scFv fragment could be complexed with either pM440-LB or pM440-SB.^17^ 1 μM of scFv was incubated overnight at 4 °C with 2 μM of pM440-LB or pM440-SB, subsequently mixed in a 1:1 ratio and diluted to 0.5 nM of scFv-LB and scFv-SB. The dose–response curve shows a 30-fold increase in bioluminescence upon increasing the concentrations of TNFα, with a lowest detected target concentration of 250 pM (Figure 6B). These results show that RAPPID-M also effectively works on antibody formats that consist solely of variable domains.

**Figure 6:**
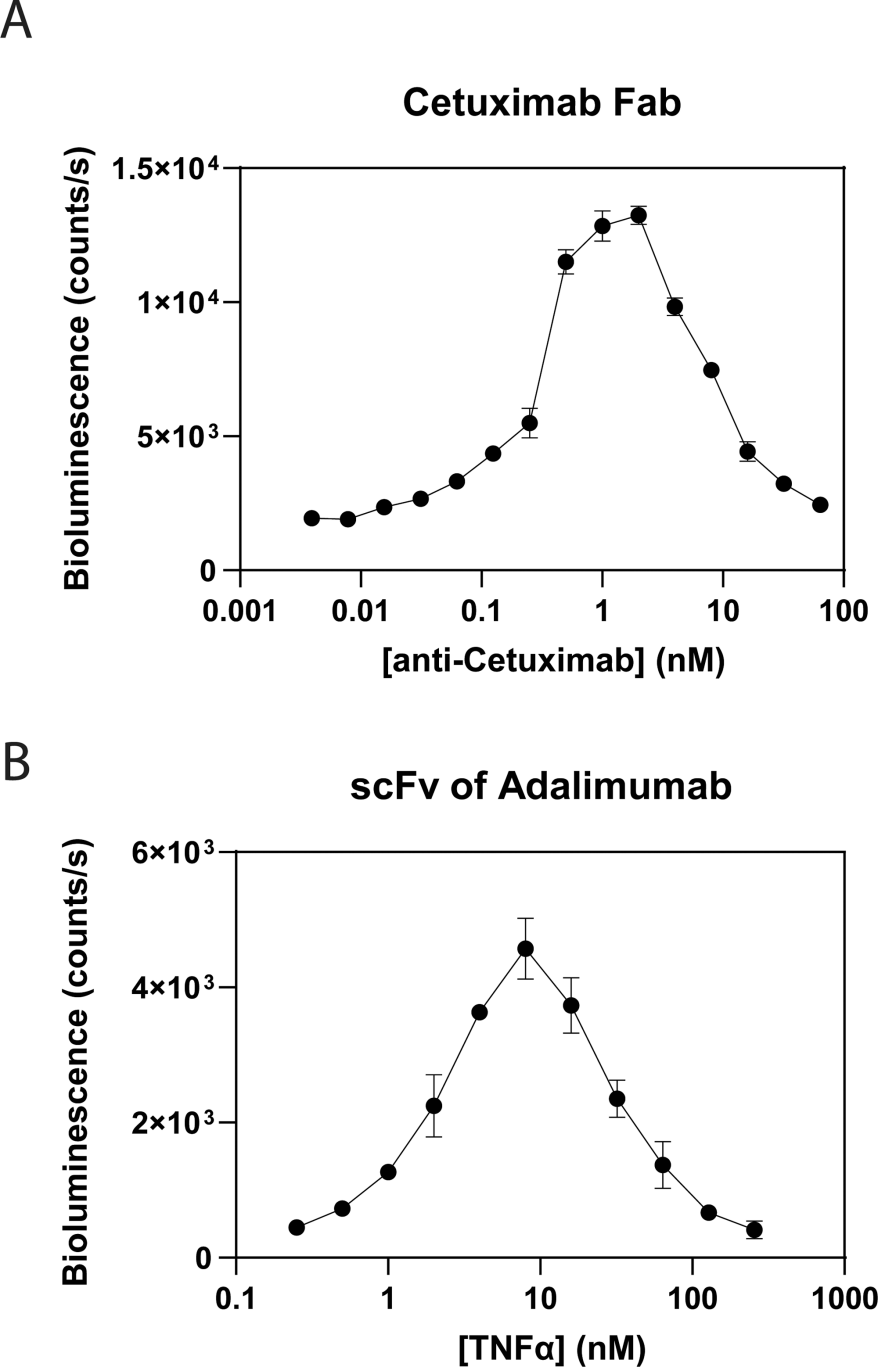
Extension of RAPPID technology to antibody fragments. (A) Dose–response of RAPPID-M sensor employing the Cetuximab Fab for the detection of anti-Cetuximab antibody. (B) Dose–response of RAPPID-M sensor employing the single-chain variable fragment for the detection of TNFα.

## Conclusions

In this study, we developed a new generation of RAPPID sensors using a truncated variant of the generic antibody binder protein M. The variant, termed protein M440, lacks the antibody-blocking domain but is found to retain the exceptionally slow dissociation kinetics of full-length protein M. As a result, RAPPID-M sensors can be readily assembled by simply mixing the protein M-fusion proteins with detection antibodies, eliminating the need for covalent photo-cross-linking mediated by protein G.^5,17,19^ The resulting complexes exhibit remarkable stability, showing minimal to no exchange upon mixing of the antibody complexes for up to 8 days.

Furthermore, the pM440-LargeBit-antibody complex displayed lower background activity compared with the pG-LB-antibody complex used in the original protein G-based RAPPID sensors. This reduction in background signal enhances the dynamic range of the RAPPID platform and lowers the LOD, enabling quantification of protein biomarkers at subpicomolar concentrations. Because protein M440 binds to the Fab region of antibodies, RAPPID-M supports a broad range of antibody types and fragments, including previously inaccessible mouse IgG1 antibodies. We also anticipate compatibility with polyclonal antibodies and avian IgY antibodies, further expanding the platform’s versatility.^23^

Sandwich assays such as RAPPID-G and RAPPID-M are not suitable for detecting small molecules, but our group recently reported a competition-based bioluminescent sensor platform for small-molecule detection called LUCOS.^8^ Like the original RAPPID sensor, the LUCOS sensor requires conjugation of a bioluminescent sensor protein to a small-molecule binding antibody via photo-cross-linking of the protein G domain. We anticipate that a protein M440-based LUCOS sensor could be engineered, abolishing the need for photochemical cross-linking and expanding the scope of suitable antibodies to include, e.g., mouse IgG1.

Beyond its current use in biosensing, truncated protein M also shows strong potential as a versatile general strategy for antibody labeling. Protein M could be prelabeled with fluorophores, radionuclides, or enzymes and complexed with antibodies without requiring postlabeling purification to remove excess probes, a common drawback of nonsite-specific methods. Additionally, the genetic fusion of truncated protein M with luminescent or fluorescent domains could expand its application to in vivo imaging. This simple, mix-and-measure approach offers an accessible, equipment-free strategy for labeling readily available antibodies with a range of functional tags.

## Material and Methods

### Molecular Cloning

The pET28a­(+) plasmid containing the protein M440-LB sequence was purchased from Genscript. To exchange the LB to SB, a PCR and LCR reaction were conducted.^28^ Briefly, 3 μM primers (forward: ACGATAGCCGGTAACaagcttgctaccacctttttc; reverse: CTGTTTGAAAAAGAGAGCggtaccggaggttcgggtggg) were phosphorylated by adding 0.2 U of T4 polynucleotide kinase (T4 PNK, NEB, #M0201S) in 1× T4 ligase buffer (NEB, #B0202S) in 50 μL and incubated for 30 min at 37 °C. The PCR mixture was prepared according to a standard protocol for Q5 high-fidelity DNA polymerase (NEB, #M0491S) by mixing the phosphorylated primers at a concentration of 480 nM with 20 ng of pM-LB plasmid and subjected to thermal cycling at the annealing temperature of 54 °C. One microliter of DpnI was added and incubated at 37 °C for 30 min. A gel-extracted product (2.77 nM) was mixed with 80 U of Taq DNA ligase (NEB, #M0208S) in 1× Taq ligase buffer in 20 μL to connect phosphorylated ends and subjected to multiple denaturation-annealing-ligation cycles using a thermostable *Taq* ligase. The reaction mixture was transformed into Top10 cells. The plasmid was extracted from a liquid culture, and the sequence was verified (BaseClear).

### Expression of Protein M Constructs

Plasmids were transformed into heat-competent *E. coli* BL21 (DE3). The overnight preculture was inoculated in a Luria broth autoinduction medium (Formedium, #AIMLB0210) supplemented with 50 μg/mL kanamycin and cultured at 37 °C for 3 h. Then, the temperature was lowered to 20 °C for an overnight expression. The cells were pelleted by centrifugation at 8000*g* for 5 min.

### Purification of Protein M Constructs

The cell pellet was dissolved in BugBuster (Novagen, #70584), 5 mL for 1 g of bacterial pellet, with the addition of 10 μL of Benzonase, and incubated for 1 h on an orbital shaker. The lysate was centrifuged for 30 min at 30,000*g* at 4 °C. The supernatant was transferred to a Ni-NTA gravity column (Qiagen, no. 30230) pre-equilibrated with Buffer A (50 mM Tris–HCl, pH 8.0, 500 mM NaCl, 10 mM imidazole, pH 7.4). The resin was washed with Buffer A and eluted with Buffer A supplemented with 350 mM imidazole. The elution was applied to a Strep-TactinXT 4Flow (IBA Lifesciences, #2–5030–025) column pre-equilibrated with Buffer B (100 mM Tris, 150 mM NaCl, 1 mM EDTA, pH 8.0), washed with the same buffer, and eluted with Buffer B supplemented with 50 mM biotin.

### Expression and Purification of scFv of Adalimumab

The pET24a plasmid with scFv insert^22^ was transformed into heat-competent BL21 (DE3)*E. coli* strain. The overnight preculture was inoculated into a Terrific Broth autoinduction medium (Formedium, #AIMTB0210) supplemented with 50 μg/mL kanamycin and cultured at 37 °C for 3 h. Then, the temperature was lowered to 18 °C for an overnight expression. The cells were pelleted by centrifugation at 8000*g* for 5 min. scFv was purified as described above for protein M constructs, with a minor change of using a hexahistidine tag only, with imidazole increased to 25 mM in Buffer A. To remove imidazole from the elution fraction, the protein was buffer exchanged using a PD-10 desalting column (Cytiva) to storage buffer (50 mM Tris–HCl, pH 8.0, 500 mM NaCl).

### Fab Fragmentation

Fab fragmentation of Cetuximab antibody was performed using the Pierce Micro Fab Preparation kit (Thermo Scientific, no. 44685) according to the included manual. Briefly, the antibody was buffer exchanged with a cysteine-containing digestion buffer. Next, the antibody was applied to a column with immobilized papain beads and incubated for 4–6 h. The column was spun to separate the resin from cleaved antibody fragments, Fab and Fc domains, which elute in the flow-through. Next, the solution was applied to a protein A column pre-equilibrated with PBS. The flow-through containing the Fab fragments was collected.

### SDS-PAGE

The samples were mixed with 4× Laemmli sample loading buffer (Bio-Rad, #1610747) and loaded into Mini-PROTEAN TGX precast gels (4–20%, Bio-Rad, #4561096 or #4561093) and run in running buffer (25 mM Tris, 192 mM glycine, 0.1% w/v SDS, pH 8.3, Bio-Rad, #1610772).

### Surface Plasmon Resonance

For the measurement of the affinity between pM440 and Adalimumab, 2 nM of the antibody diluted in the running buffer (10 mM HEPES, pH 7.5, 150 mM NaCl, 3 mM EDTA, 0.005% P20 surfactant (cytiva, #BR100054)) was flown over the protein G-functionalized chip (cytiva, #29179316). Next, pM440 was flown over at a rate of 30 μL/min for 180 s, followed by the dissociation for 1 h with the running buffer. The chip was regenerated by applying 10 mM glycine of pH 1.5 in pulses of 30 and 10 s. For the measurement of the affinity between Adalimumab and TNFα, 3 nM antibody or an overnight incubated antibody-pM440 (1 μM antibody, 1:4 ratio) complex was flown over the chip in the running buffer. Subsequently, the dissociation step was conducted by flushing the running buffer over the chip for 600 s. Typically, the regeneration was conducted by flushing 10 mM glycine, pH 1.5, over the chip in two pulses of 30 and 10 s. All measurements were performed on Biacore ×100 with a flow rate of 30 μL/min. The obtained sensograms were analyzed in the Biacore Evaluation software by removing the bulk shift data points and fitting the blank-subtracted measurements in the 1:1 binding model.

### Comparison of RAPPID-G and RAPPID-M

RAPPID-M components were prepared by mixing 1 μM R508 and 1 μM mhK23 antibodies with pM440-LB and pM440-SB, respectively, in a 1:2 ratio and incubating overnight at 4 °C. RAPPID-G components were prepared by mixing 1 μM R508 and 1 μM mhK23 antibodies with pG-LB and pG-SB, respectively, in a 1:2 ratio, incubating for 45 min, and irradiating with UV light for 10 min. RAPPID-G and RAPPID-M were diluted to 0.1 nM R508-LB and 0.5 nM mhK23-SB, respectively. The sensor components were mixed with different concentrations of IL-6 in buffer (PBS, pH 7.4, 1% (w/v) BSA) and incubated for 2 h at RT. Just before the measurement, 500× diluted NGlo was added and measured on a SPARK plate reader (Tecan) using a luminescence module, with an integration time of 1000 ms. The limit of detection (LOD) was calculated as the signal of the blank plus 3 times the standard deviation of the blank. The first concentration measured above this value is reported.

### Background Characterization of pM- and pG-Based RAPPID

The RAPPID sensor components were prepared as described above. The probes were diluted to 0.1 nM R508-LB and 0.5 nM mhK23-SB (separately or combined) in a buffer (PBS, pH 7.4, 1% (w/v) BSA) and incubated for 2 h. 500× diluted Nano-Glo was added, and the samples were measured on a SPARK plate reader (Tecan) using a luminescence module, with an integration time of 1000 ms.

### RAPPID-M Assays

Typically, protein M440 was mixed with an antibody or antibody fragment in a ratio of 1:2 (unless stated otherwise) and incubated overnight. The next day, the complexes were mixed and diluted to 0.1–0.5 nM with either a 1:1 or 1:5 ratio of LB:SB in PBS with the addition of 0.1 or 1% BSA. The sensor mixture was mixed with various concentrations of analyte, incubated for 1 to 2 h (or overnight). Then, 500–1000× diluted Nano-Glo substrate was added, and the samples were measured on a SPARK plate reader (Tecan) using a luminescence module, with an integration time of 100 or 1000 ms. The limit of detection (LOD) was calculated as the signal of the blank plus 3 times the standard deviation of the blank. The first measured concentration above this value is reported.

## Supplementary Material



## Data Availability

The authors will share all raw data produced in this study upon request.

## Notes

Additional materials and methods, supporting figures, and protein sequences (PDF)
