# Supplementary material for: RAPPID-M: A Mix-and-Measure Bioluminescent Sandwich Immunoassay Based on Generic Antibody-Binding Protein M
Source: ACS Sens. 2025 Oct 10;10(10):7915–23. doi: 10.1021/acssensors.5c02450 (PMC12560131; doi:10.1021/acssensors.5c02450)
Supplement: Supplementary file 1 [file se5c02450_si_001.pdf]

RAPPID-M : a mix-and-measure bioluminescent sandwich immunoassay based on generic antibody binding protein M.

Anna Swietlikowska[ab], Eva van Aalen[ab] Max Bossink[ab], Laura van Weesep[ab], Maarten Merkx[ab *]

a Laboratory of Chemical Biology, Department of Biomedical Engineering, PO box 513, 5600 MB, Eindhoven University of Technology, Eindhoven, The Netherlands

b Institute for Complex Molecular Systems (ICMS), Eindhoven University of Technology, PO box 513, 5600 MB, Eindhoven, The Netherlands

* Corresponding author: Maarten Merkx, m.merkx@tue.nl

Table of contents: Page

Supplementary Methods:

	Differential scanning fluorimetry (DSF)	S3
	Size exclusion chromatography (SEC)	S3
	Custom single cycle SPR measurements	S3
	Influence of sensor incubation time	S3
	Sensor performance in the presence of human serum	S3

Supplementary Figures:

Figure S1	Protein sequences	S4
Figure S2	Reducing SDS-PAGE displaying purifications of utilized proteins	S5
Figure S3	Differential scanning fluorimetry (DSF)	S6
Figure S4	Size exclusion chromatography (SEC) of protein M-antibody complexes	S6
Figure S5	Fitted SPR sensogram shown in Figure 2a	S7
Figure S6	Fitted SPR sensograms shown in Figure 2b-c	S7
Figure S7	SPR sensorgram displaying the saturation of an antibody with pM440 for single cycle measurement.	S8
Figure S8	SPR analysis of affinity between an Adalimumab-TNFα union in the absence and presence of pM440 or the CDR blocking variant pM468	S9
Figure S9	SPR analysis of affinity between an R508-IL-6 union in the absence and presence of pM440	S10
Figure S10	Influence of sensor incubation time	S11
Figure S11	Sensor performance in the presence of human serum	S11
Figure S12	Purified scFv of Adalimumab	S12
	Supporting Information references	S12

Supplementary methods:

Differential scanning fluorimetry (DSF). 5 µM protein was mixed with 5x concentrated ProteoOrange® dye (Lumiprobe, 5000x stock in DMSO) in PBS and heated from 25 to 90 °C at the rate of 0.3 °C/15 s in a Biorad CFX96 Touch Real-Time PCR.

Size exclusion chromatography (SEC). 0.75 mg of pM440 (33.33 µM) or 0.375 mg of Adalimumab (5 µM) or their complex were applied to 24 mL ENrich™ 650 10 x 300 SEC column (Biorad, #7801650). The runs were performed in 50 mM Tris, pH 7.5, 150 mM NaCl using a flow of 0.5 mL/min.

Custom single cycle SPR measurements. An antibody was immobilized on a protein G chip (cytiva, #29179316). Subsequently, protein M variants (or buffer) were introduced till the antibody was saturated. For Adalimumab, 2 nM was immobilized for 30 s at a rate of 30 µL/min for 180 s, followed by saturation of 1.29 µM of protein M variants (Figure S7), and subsequently the analyte in concentrations of 0.25-64 nM (4 fold step dilution) was flown over. For R508, 10 nM was immobilized for 30 s at a rate of 30 µL/min for 180 s, followed by saturation of 2 µM of protein M variants and the concentrations of 0.2-16.2 (3 fold step dilution) were flown over. Next, the step of 9 min (Adalimumab) or 30 min (R508) dissociation followed. The bulk shift jumps caused by refractive index difference were removed from the blank subtracted sensorgrams and the affinity of the antibody for TNFα or IL-6 was calculated with Biacore Evaluation software using 1:1 kinetic binding model.

Influence of sensor incubation time. Anti-IL-6 RAPPID-M sensors were prepared by mixing 1 µM of pM440-LB or pM440-SB with R508 or mhK23 antibodies (1:2 ratio), respectively and incubated overnight at 4 °C or prepared directly before the assay set-up. RAPPID-M components were diluted to 0.2 nM R508-LB and 1 nM mhK23-SB. The sensor components were mixed with different concentrations of IL-6 in buffer (PBS, pH 7.4, 1% (w/v) BSA), incubated for 1.5 hour at RT. Just before the measurement, 1000x diluted NGlo (Promega, #N1110) was added and the samples were measured on a SPARK® plate reader (Tecan) using luminescence module, with integration time of 100 ms.

Sensor performance in the presence of human serum. Anti-IL-6 RAPPID-M sensors were prepared by mixing 1 µM of pM440-LB or pM440-SB with R508 and mhK23 antibodies (1:2 ratio), respectively, and kept at 4 °C. RAPPID-M components were diluted to 0.2 nM R508-LB and 1 nM mhK23-SB. The sensor components were mixed with different concentrations of IL-6 or IL-6 supplemented with 10% human serum (Sigma, #S2257) in buffer (PBS, pH 7.4, 1% (w/v) BSA) and incubated for 2 hours at RT. 1000x diluted NGlo substrate (Promega, #N1110) was added and the samples were measured on a SPARK® plate reader (Tecan) using luminescence module, with integration time of 100 ms.

Supplementary Figures:

Protein M440 sequence:
MGSSHHHHHHKLSGTLVPRGSHMSLSL**NDGSYQSEIDLSGGANFREKFRNFANEL SEAITNSPKGLDRPVPKTEISGLIKTGDNFITPSFKAGYYDHVASDGSLLSYYQSTEY FNNRVLMPILQTTNGTLMANNRGYDDVFRQVPSFSGWSNTKATTVSTSNNLTYDK WTYFAAKGSPLYDSYPNHFFEDVKTLAIDAKDISALKTTIDSEKPTYLIIRGLSGNGS QLNELQLPESVKKVSLYGDYTGVNVAKQIFANVVELEFYSTSKANSFGFNPLVLGS KTNVIYDLFASKPFTHIDLTQVTLQNSDNSAIDANKLKQAVGDIYNYRRFERQFQGY FAGGYIDKYLVKNVNTNKDSDDDLVYRSLKELNLHLEEAYREGDNTYYRVN**EFGGS GGSWSHPQFEKC

Sequence of pM-440-LB:
MGWSHPQFEKGGSKLVFTLEDFVGDWEQTAAYNLDQVLEQGGVSSLLQNLAVSVT PIQRIVRSGENALKIDIHVIIPYEGLSADQMAQIEEVFKVVYPVDDHHFKVILPYGTLVID GVTPNMLNYFGRPYEGIAVFDGKKITVTGTLWNGNKIIDERLITPDGSMLFRVTINGT GGSGGSGGSGGSGGSGGSGEFAEAAAKEAAAKEAAAKEAAAKEAAAKEAAAKAEF GGSGGSGGSGGSGGSGGSGGTGCGGSLSL**NDGSYQSEIDLSGGANFREKFRNF ANELSEAITNSPKGLDRPVPKTEISGLIKTGDNFITPSFKAGYYDHVASDGSLLSYY QSTEYFNNRVLMPILQTTNGTLMANNRGYDDVFRQVPSFSGWSNTKATTVSTSNN LTYDKWTYFAAKGSPLYDSYPNHFFEDVKTLAIDAKDISALKTTIDSEKPTYLIIRGLS GNGSQLNELQLPESVKKVSLYGDYTGVNVAKQIFANVVELEFYSTSKANSFGFNPL VLGSKTNVIYDLFASKPFTHIDLTQVTLQNSDNSAIDANKLKQAVGDIYNYRRFERQ FQGYFAGGYIDKYLVKNVNTNKDSDDDLVYRSLKELNLHLEEAYREGDNTYYRVN**E FGSAAALEHHHHHH

Sequence of pM440-SB:
MGWSHPQFEKGGSKLVTGYRLFEKESGTGGSGGSGGSGGSGGSGGSGEFAEAA AKEAAAKEAAAKEAAAKEAAAKEAAAKAEFGGSGGSGGSGGSGGSGGSGGTGCG GSLSL**NDGSYQSEIDLSGGANFREKFRNFANELSEAITNSPKGLDRPVPKTEISGLI KTGDNFITPSFKAGYYDHVASDGSLLSYYQSTEYFNNRVLMPILQTTNGTLMANNR GYDDVFRQVPSFSGWSNTKATTVSTSNNLTYDKWTYFAAKGSPLYDSYPNHFFED VKTLAIDAKDISALKTTIDSEKPTYLIIRGLSGNGSQLNELQLPESVKKVSLYGDYTG VNVAKQIFANVVELEFYSTSKANSFGFNPLVLGSKTNVIYDLFASKPFTHIDLTQVTL QNSDNSAIDANKLKQAVGDIYNYRRFERQFQGYFAGGYIDKYLVKNVNTNKDSDD DLVYRSLKELNLHLEEAYREGDNTYYRVN**EFGSAAALEHHHHHH

Figure S1 Protein sequences of a) pM440, b) LB-pM440, c) SB-pM440. The protein M sequences are shown in bold and the additional colored sequences are hexa-histidine tag (magenta), strep tag (purple) and sequences of LargeBit and SmallBit (shaded in blue).

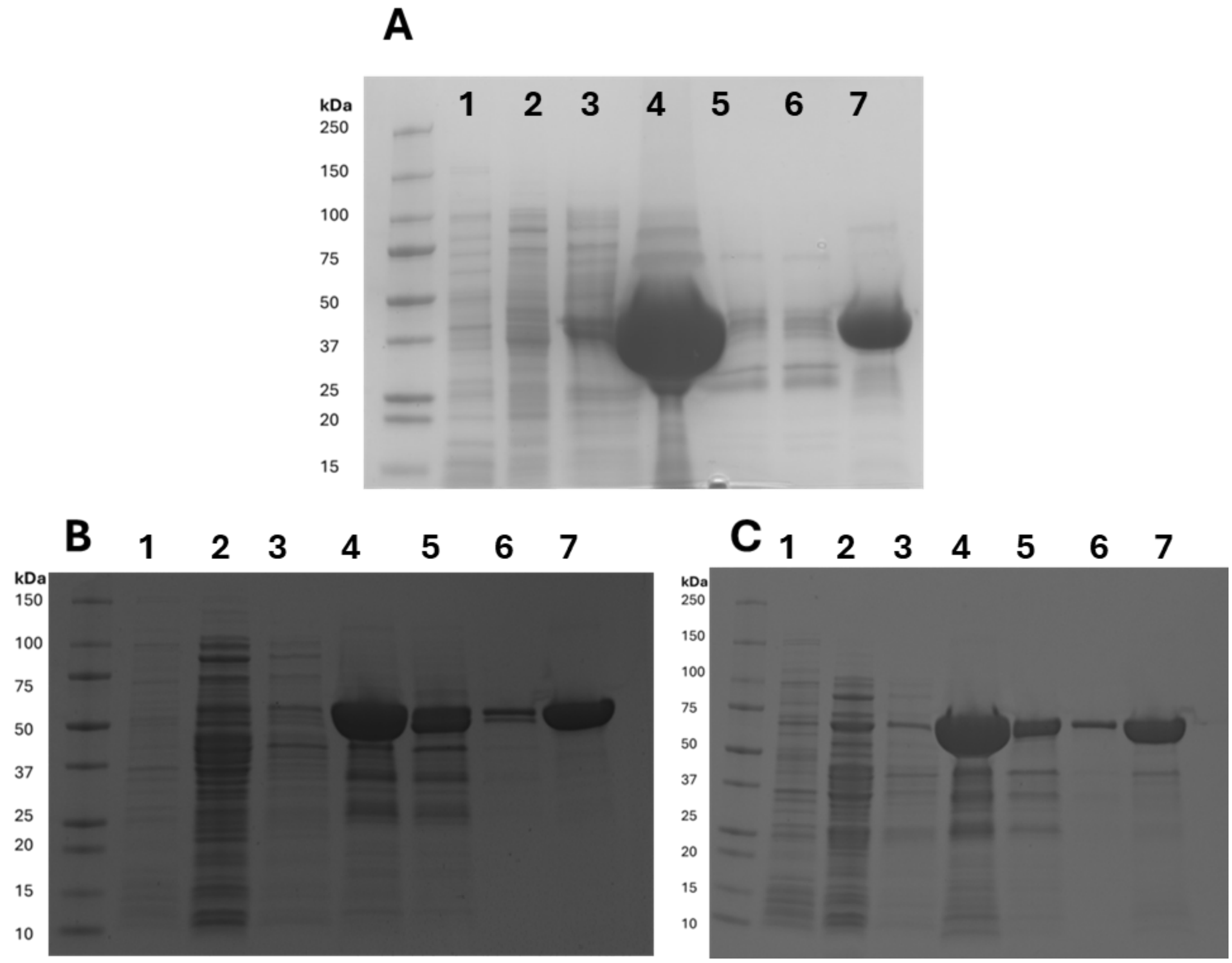

Figure S2 Reducing SDS-PAGE displaying purification of A) pM440, B) pM440-SB and C) pM440-LB. The lanes are as follows: 1 Pellet (insoluble fraction), 2. Flow through his-tag purification, 3 wash his-tag purification, 4 elution his-tag purification, 5 flow through strep-tag purification, 6. Wash strep-tag purification, 7 elution strep-tag purification.

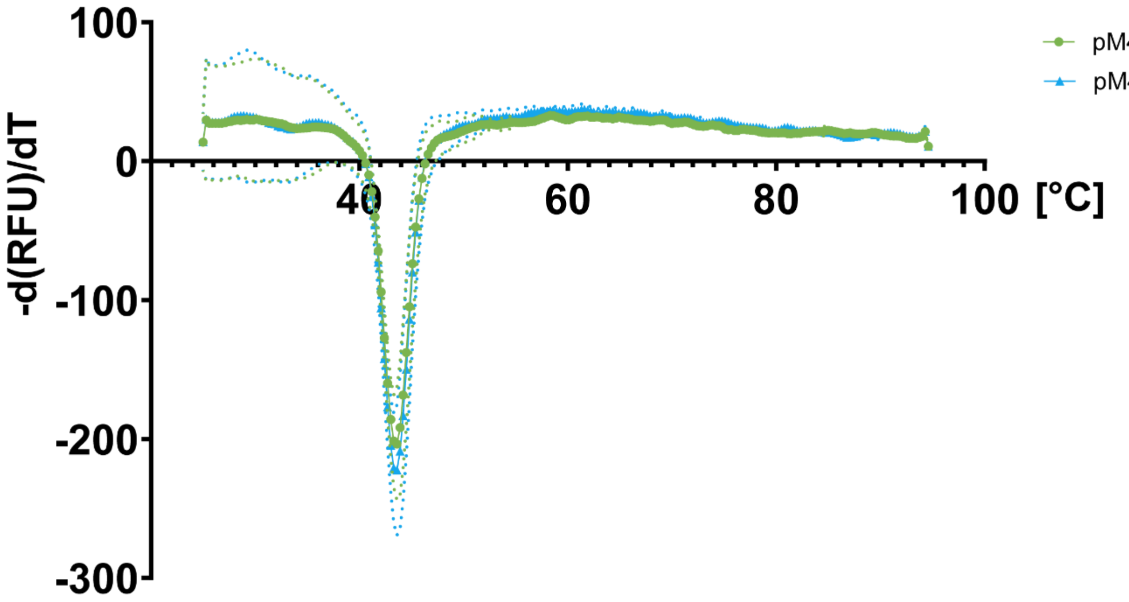

Figure S3 Differential scanning fluorimetry (DSF). The melting temperature of the full length protein (pM 468) was 43.8 °C and of the protein M440 was 43.5 °C. The signal shown as the inverse of the first derivative of the fluorescence signal and the graph displays the mean of triplicates with the error bars displayed as dashed line.

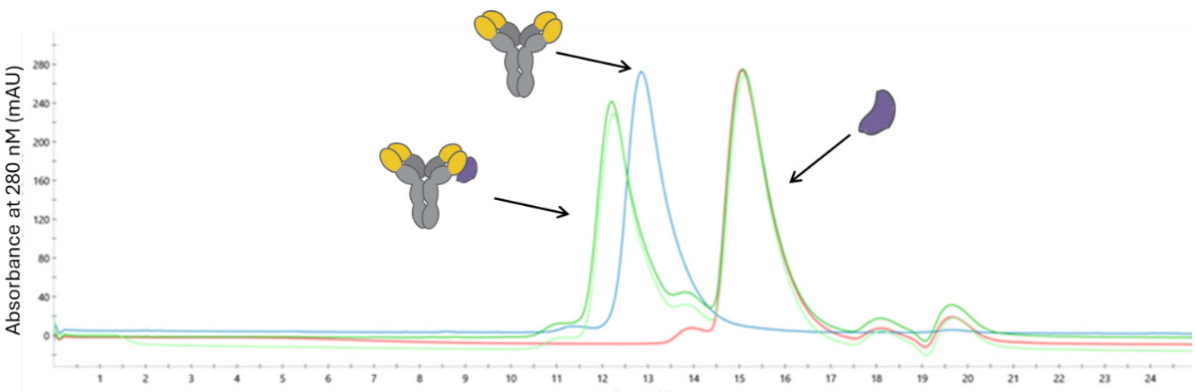

Figure S4 Size exclusion chromatography (SEC) of protein M-antibody complexes. 0.75 mg of pM440 or 0.375 mg of Adalimumab or the complex thereof were applied to Enrich™ 650 10 x 300 SEC column. pM440 (red) and Adalimumab (blue) are respectively 45 kDa and 150 kDa. The formed complex is eluting faster due to its increased mass. Both measurements for 15 min incubation (light green) and overnight incubation (dark green) overlap, showing that 15 min is enough to form the complex.

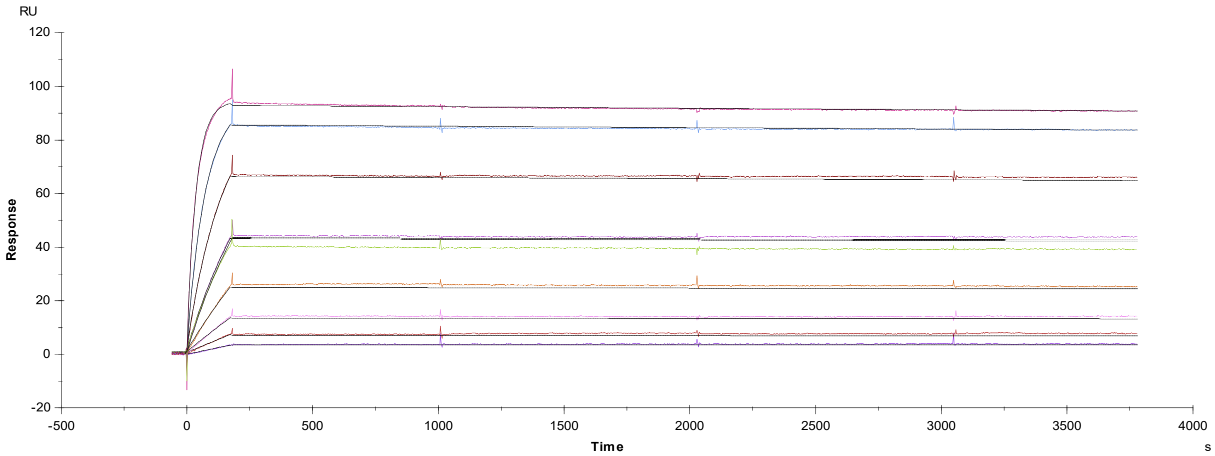

Figure S5 Fitted SPR sensorgrams displaying the binding of the pM440 to Adalimumab shown in Figure 2a. 2 nM of Adalimumab diluted in the running buffer (10 mM HEPES, pH 7.5, 150 mM NaCl, 3 mM EDTA, 0.005% P20 surfactant (cytiva, #BR100054)) was immobilized on the protein G functionalized chip (cytiva, #29179316). pM440 was flown over the chip in the concentrations ranging from to 5 nM to 640 nM (2 fold step dilution). Fitting of the blank subtracted sensorgram with 1:1 binding model was obtained and analysed with Biacore Evaluation software.

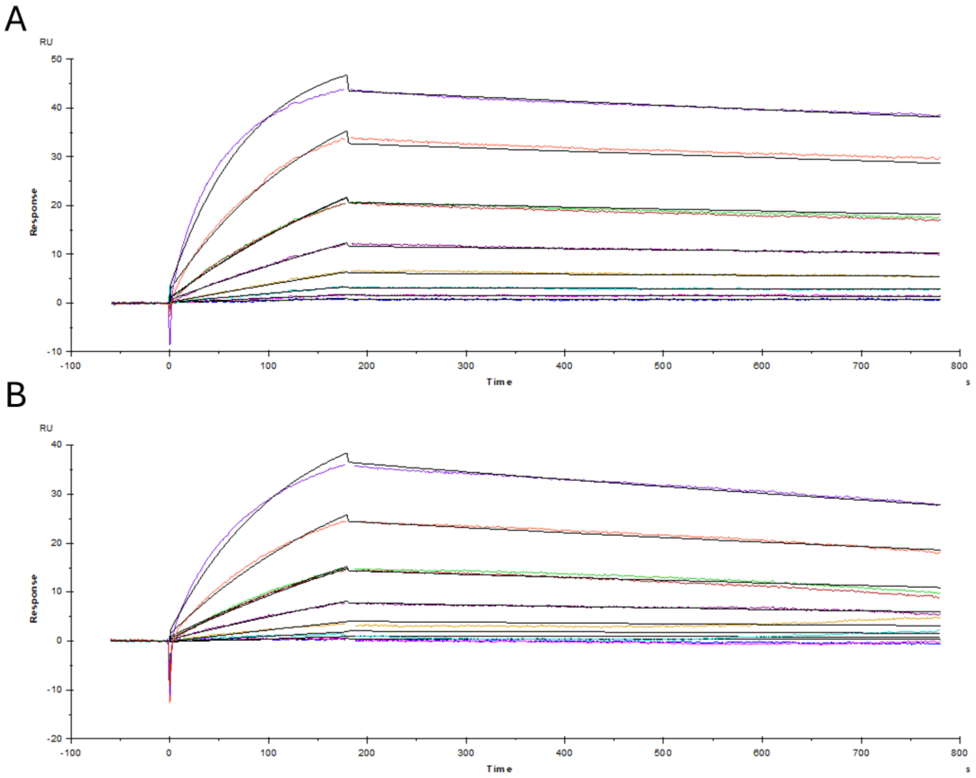

Figure S6 Fitted SPR sensorgrams displaying the binding of the TNFα to Adalimumab (S6a) or to Adalimumab-pM440 complex (S6b) shown in Figure 2b-c. Adalimumab was preincubated with pM440 (ratio 1:4) overnight at 4°C. 3 nM of Adalimumab or Adalimumab-pM440 was flushed over protein G chip. After the immobilization, TNFα was flown over the chip in the range of concentrations from 0.39 nM to 12.5 nM. Fitting of the blank subtracted sensorgram was obtained with 1:1 binding model and analysed with Biacore Evaluation software.

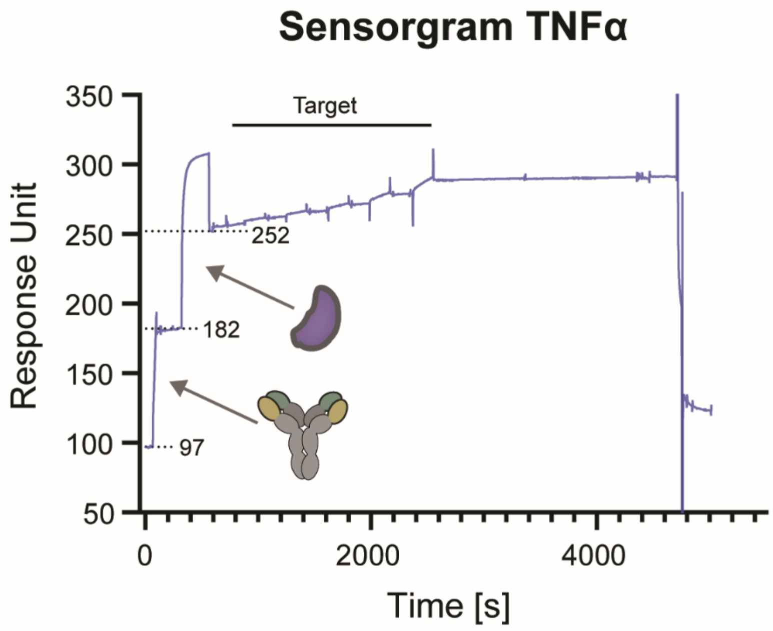

Figure S7 SPR sensorgram displaying the saturation of an antibody with pM440 for single cycle measurement. The antibody was applied to a protein G chip and subsequently saturated with pM440 leading to saturation of the antibody's light chains. Finally, the increasing concentrations of the analyte were flushed over the chip, each followed by the short dissociation step, followed by the final long dissociation step of 9 min. The response unit increases are consistent with the binding of 2 pM440 domains per antibody.

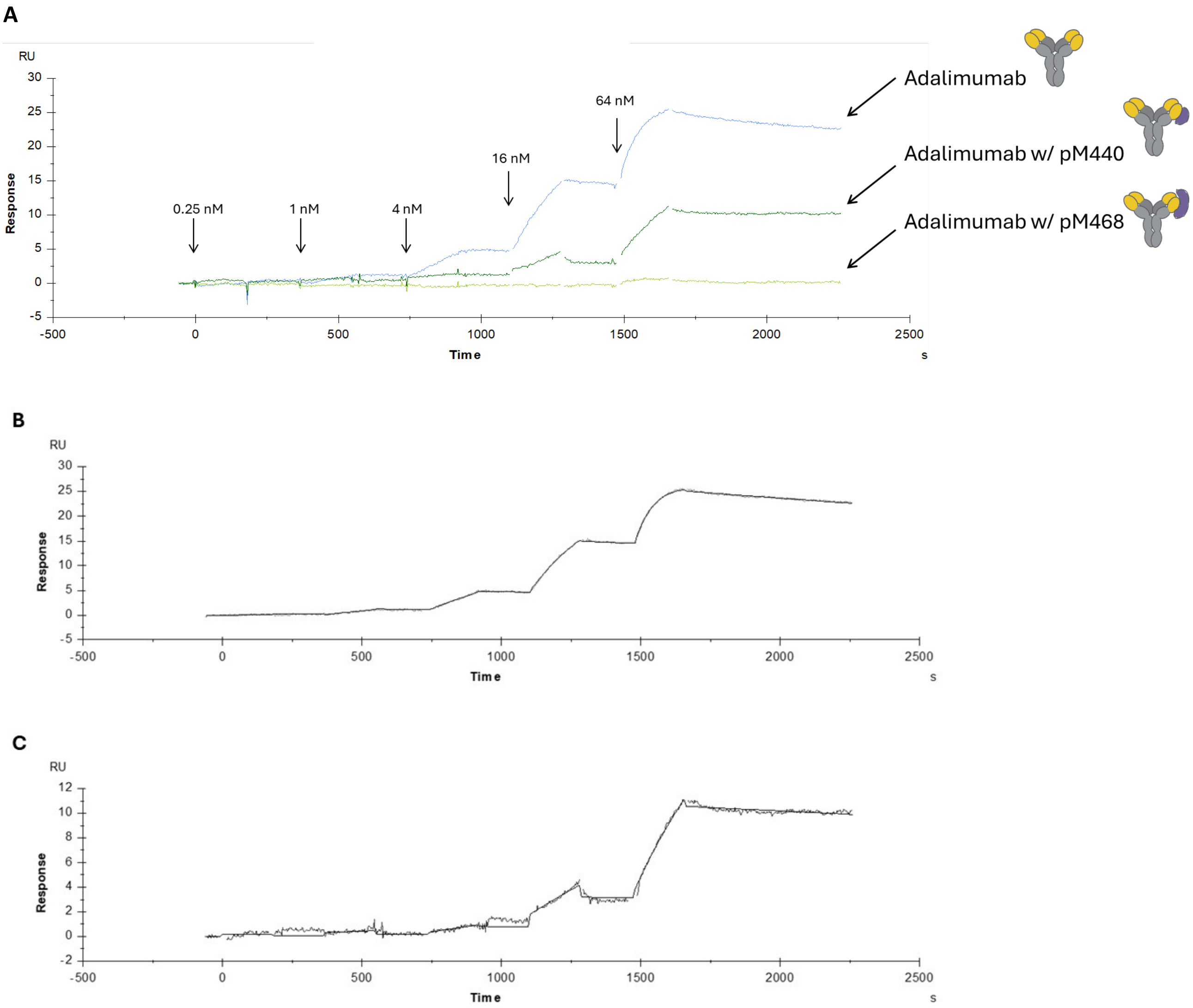

D

	Adalimumab-TNFα affinity						
	K_a (1/Ms)	K_{diss} (1/s)	KD (M)	R_{max} (RU)	tc	Chi² (RU²)	U-value
**No protein M**	2,73 $\times 10^{5}$	1,76 $\times 10^{-4}$	6,45$\times 10^{-10}$	25,85	9,33 $\times 10^{6}$	0,0235	1
**Protein M440**	3,59 $\times 10^{4}$	1,05 $\times 10^{-4}$	2,94 $\times 10^{-9}$	25,40	4,63 $\times 10^{10}$	0,0737	7

Figure S8 SPR analysis of binding between an Adalimumab and TNFα in the absence and presence of pM440 or the CDR blocking variant pM468. The antibody was applied to a protein G chip. Then, the various protein M variants (or buffer) were flushed over the chip, leading to saturation of the antibody's light chains. Five concentrations of TNFα were flushed over the chip in a single cycle kinetic titration cycle. A) Combined blank subtracted sensorgrams of Adalimumab-TNFα interaction in the absence and in the presence of pM440 and pM468. The data fits based on 1:1 binding model are shown in B) for Adalimumab and TNFα interaction and C) for Adalimumab-pM440 and TNFα interaction. D) The affinity analysis of the fitted sensorgrams and fitting validation data obtained using Biacore Evaluation software.

A

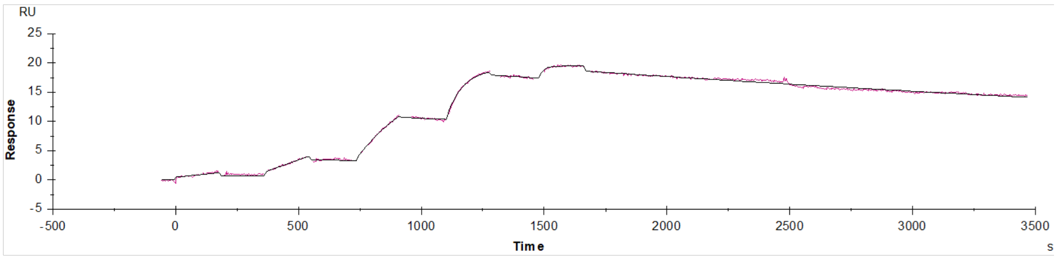

B

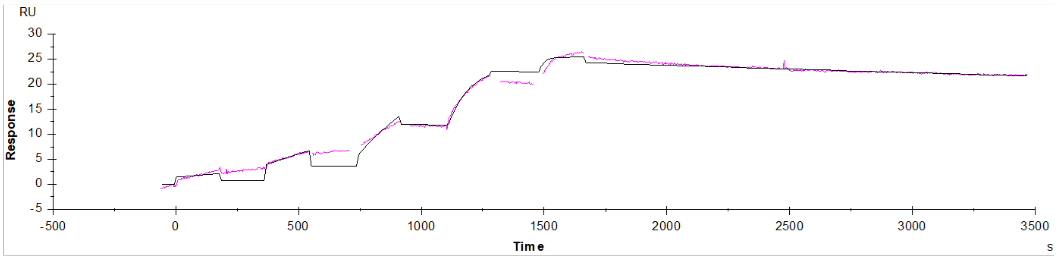

C

	R508-IL-6						
	K_a (1/Ms)	K_{diss} (1/s)	KD (M)	R_{max} (RU)	tc	Chi² (RU²)	U-value
**No protein M**	9,33 $\times 10^5$	1,53 $\times 10^{-4}$	1,64 $\times 10^{-10}$	18,68	6,20 $\times 10^{17}$	0,0444	1
**Protein M440**	7,26 $\times 10^5$	6,43 $\times 10^{-5}$	8,85 $\times 10^{-11}$	24,31	8,85 $\times 10^{16}$	0,976	7

Figure S9 SPR analysis of affinity between an R508 (Sino Biological, #10395-R508) and IL-6 (Sigma, #SCU0001) union in the absence and presence of pM440. R508 antibody was immobilised on protein G functionalised chip and subsequently saturated with protein M variant (or buffer). Five concentrations of IL-6 ranging of 0.2-16.2 nM (3 fold step dilution) were flushed over the chip in a single cycle kinetic titration cycle followed by the dissociation of 1 h in running buffer. The data fits based on 1:1 binding model are shown in A) for R508-IL-6 interaction displaying the dissociation constant of 160 pM, B) R508-pM440-IL-6 interaction displaying the dissociation constant of 89 pM, C) The affinity analysis of the fitted sensogram and fitting validation data obtained using Biacore Evaluation software. Noteworthy, the estimated dissociation constants for the studied interaction are reaching the detection limit of Biacore X100 of 100 pM.

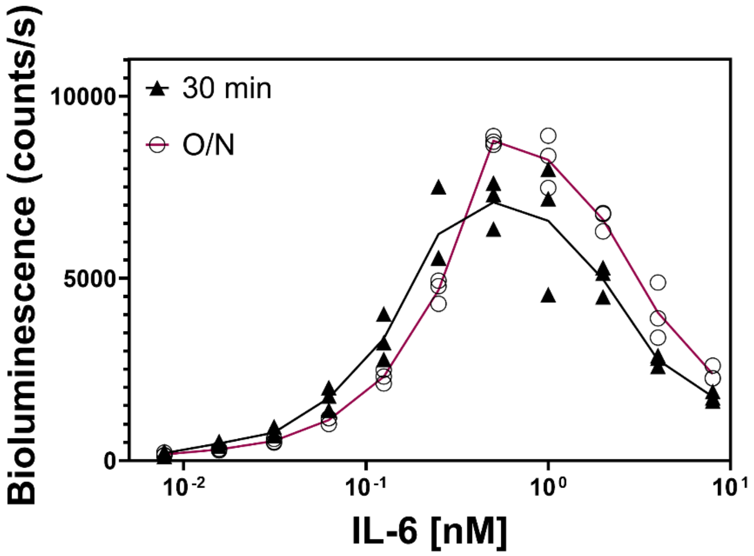

Figure S10 Influence of sensor incubation time. Dose response curves of RAPPID-M sensors prepared either by overnight or 30 min incubation. RAPPID-M sensors were prepared by mixing 1 µM of pM440-LB or pM440-SB with R508 or mhK23 antibodies (1:2 ratio), and incubated overnight at 4°C or for 30 min. Sensors were diluted to 0.2 nM pM440-LB-R508 and 1 nM pM440-SB-mhK23 and mixed with increasing concentration of interleukin-6 (IL-6). Both sensors displayed similar characteristics, with extremely low background (14 and 23 counts/s), both measured increasing concentration of IL-6, showed maximum signal at 500 pM of IL-6 and displayed large fold change of 506x and 387x for 30 min and overnight, respectively.

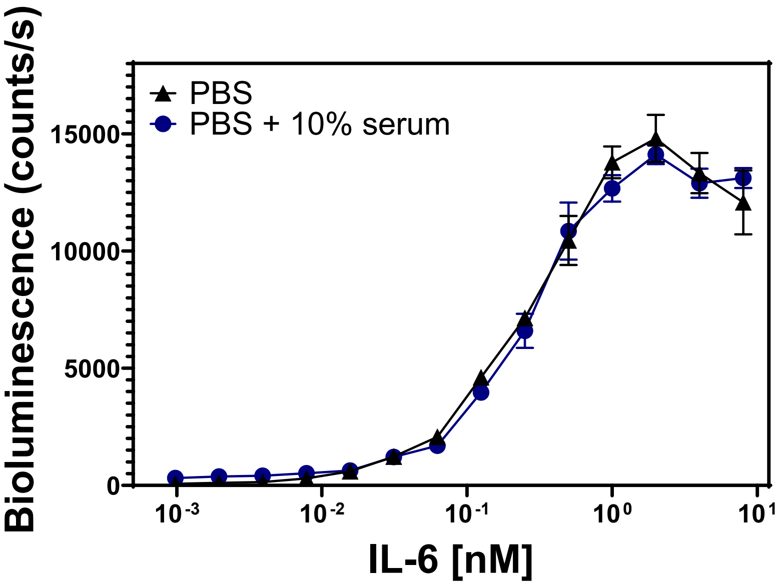

Figure S11 Sensor performance in the presence of human serum. Dose response curves for RAPPID-M sensors were measured in samples containing 10% serum. Sensors were prepared by mixing 1 µM of pM440-LB or pM440-SB with R508 or mhK23 antibodies (1:2 ratio), and stored at 4°C. Prior to the measurement, sensors were diluted to 0.2 nM pM440-LB-R508 and 1 nM pM440-SB-mhK23 and mixed with increasing concentration of interleukin-6 (IL-6) or IL-6 supplemented with 10% serum.

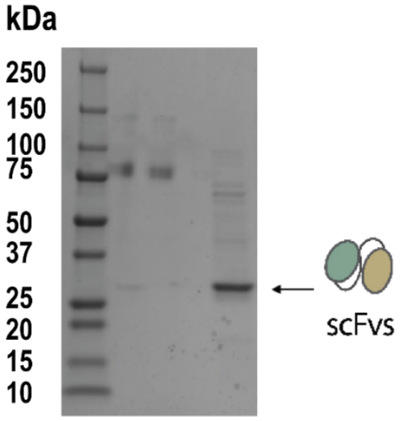

Figure S12 Purified scFv of Adalimumab. The sequence and the purification method were followed as described previously. [1]

References:

1. Biewenga, L., Vermathen, R., Rosier, B. J. H. M., & Merkx, M. (2024). A Generic Antibody-Blocking Protein That Enables pH-Switchable Activation of Antibody Activity. *ACS Chemical Biology*, *19*(1), 48–57.
